# Using transect sampling to determine the distribution of some key non-timber forest products across habitat types near Boumba-Bek National Park, South-east Cameroon

**DOI:** 10.1186/s12898-019-0219-y

**Published:** 2019-01-22

**Authors:** T. Marlène Ngansop, Elvire H. Biye, F. Evariste Fongnzossie, Preasious F. Forbi, D. Cédric Chimi

**Affiliations:** 1 Department of Plant Biology, Faculty of Science, University of Yaoundé I, P.O. Box: 812, Yaoundé, Cameroon; 2 Higher Teacher’s Training School for Technical Education (ENSET), University of Douala, P.O. Box: 1872, Douala, Cameroon; 3 Institute of Agricultural Research for Development (IRAD), P.O. Box: 230, Bertoua, Cameroon

**Keywords:** Forest resources, NTFPs, Exploitation, Sustainability, Human activities

## Abstract

**Background:**

Understanding the variation in distribution and abundance of non-timber forest products (NTFP) species is a crucial step in achieving their conservation and sustainable use. At the northern periphery of the Boumba-Bek National Park in Southeast Cameroon, little is known about which habitat type contain the highest abundance of NTFP species. In this study, we assessed habitat diversity and variation in the abundance of eight priority NTFP species comprising: *Afrostyrax lepidophyllus, Baillonella toxisperma*, *Irvingia gabonensis, Panda oleosa, Pentaclethra macrophylla*, *Ricinodendron heudelotii*, *Scorodophloeus zenkeri* and *Tetrapleura tetraptera*. The inventory was done along 16 linear transects of 5000 m × 20 m, and all the individuals, from juveniles (DBH < 5 cm) to mature trees (DBH > 5 cm) of the eight NTFPs were recorded. Habitat types were characterized along transects following basic forest classification system used in ecology and then measured.

**Results:**

In total, 13 different habitat types were identified with young secondary forests and periodically flooded forests representing 32.70% and 26.31% respectively. The least represented habitat was young fallows with *Chromolaena odorata* (0.08%). Seven NTFPs (*A. lepidophyllus*, *B. toxisperma*, *I. gabonensis*, *P. oleosa*, *P. macrophylla*, *R. heudelotii* and *T. tetraptera*) were predominantly represented in young secondary forests whereas *S. zenkeri* was more abundant in young Marantaceae secondary forests. The different types of young secondary forests identified seem to be favourable for the growth of the eight NTFPs.

**Conclusions:**

This study demonstrated that habitat fragmentation driven by human activities such as industrial logging and shifting cultivation destroy the forest ecosystems and has a strong influence on the sustainability of the major NTFPs in the locality.

**Electronic supplementary material:**

The online version of this article (10.1186/s12898-019-0219-y) contains supplementary material, which is available to authorized users.

## Background

The tropical forest of Cameroon is divided into three main groups of ecosystems namely: coastal lowlands, evergreen and semi-deciduous tropical forests [1]. As a result of such diversity, the country is often said to be “Africa in miniature” [2]. These forest ecosystems provide a variety of goods and services that are vital for subsistence and livelihood of millions of local populations who rely on them for subsistence uses, cash income, or both [3]. The country’s strategy to improve the contribution of forests in poverty alleviation has put great emphasis on the development of value chains of non timber forest products (NTFPs). These products are also presented as viable solutions to improve the livelihood of forest dependent communities while ensuring forest conservation. When NTFPs move from subsistence use to commercialized products, the livelihoods of harvesters, collectors, traders, transporters, wholesalers and consumers become interlinked.

Despite their potentials, the management of NTFPs is currently hampered by poor knowledge of species in terms of distribution, population status and productivity. As a result, ecological assessments of NTFPs are of fundamental importance to forest management. They provide information on the distribution pattern of key species on which harvesting plans can be developed [4].

Several socio economic and botanical studies have been realized within and around the Boumba-Bek National Park. In the Gribe village for example, wild fruits contribute significantly to the household economy and NTFPs account for 70% of the total number of sales cases, representing net gains of approximately 5,200,000 XFA annually (10,000 USD) [5]. The species targeted by this study have been investigated for their natural regeneration capacity in the study area and it was reported that *Baillonella toxisperma* Pierre (Moabi), *Irvingia gabonensis* (Aubry. Lecomte ex O. Rorke) Bail. (Bush mango), *Panda oleosa* Pierre (Bush groundnut), and *Tetrapleura tetraptera* (Thonn.) Taub. (Aidan tree) have lower natural regeneration potentials, while *Afrostyrax lepidophyllus* (Harms) Mildbr. (Garlic tree), *Pentaclethra macrophylla* Benth. (African oil bean), *Ricinodendron heudelotii* (Bail.) Pierre ex Heckel (Njansang) and *Scorodophloeus zenkeri* Harms (Garlic tree) possess higher natural regeneration potentials [6]. Evidence of high habitat diversity has been reported in previous studies in this area [7]. However, the distribution patterns of selected key NTFPs species exploited in the area are still not understood, both within and across habitat types.

The sustainability of livelihood of population in Protected Area landscapes remains a major preoccupation because the creation of protected areas regulates access to forest resources, depriving the forest inhabitants of full benefits from the user rights for their subsistence compared to what they had as benefits before the creation of the Protected Area. This leads to the intensive use of the available resources and the diversification in land use types near Protected Areas [8]. Thus, the vegetation cover as well as the availability of species providing NTFPs is progressively deteriorating, in particular with regards to their irrational use [9]. There is therefore the need to understand the impacts of different anthropic disturbances on the dynamics and functioning of forests, and thus the abundance and distribution of NTFP species as the basis for any sustainable NTFP exploitation [10].

The objectives of this study were to (1) survey habitat diversity, and (2) assess the population distribution of the selected NTFPs across the different habitat types in the rainforests at the northern periphery of the Boumba-Bek National Park (eastern Cameroon).

## Materials and methods

### Study site

**Fig. 1:**
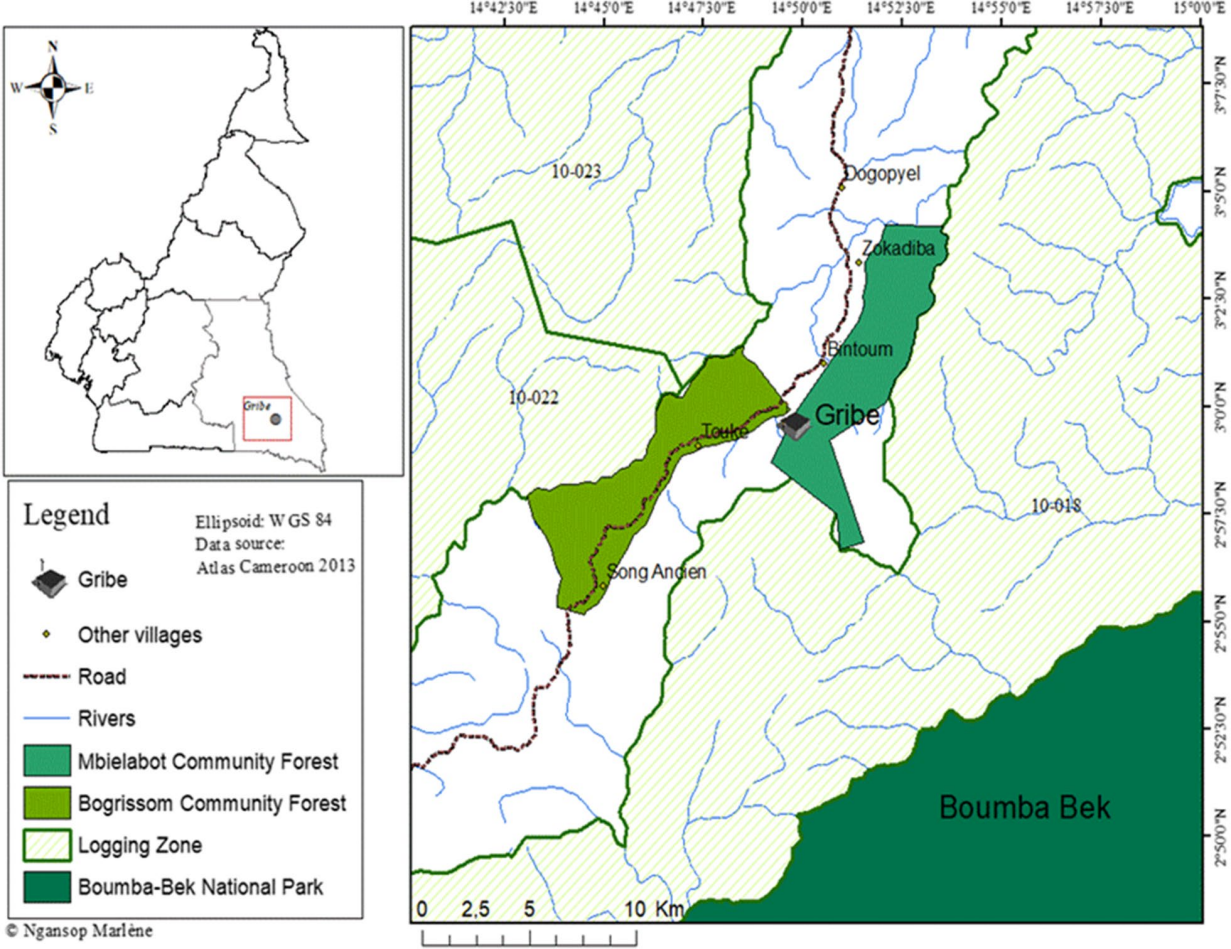
Study area

The study was carried out in the Gribe village, found in the Yokadouma sub-division of the Boumba and Ngoko Division in the East region of Cameroon. It is localized at latitude 03°00′10″N and longitude 14°49′25″E. Gribe is located about 76 km south-west of Yokadouma town and 16.5 km northeast of Boumba-Bek National Park. It extends on about 12 km, with a population of approximately 772 inhabitants [11]. Several ethnic groups constitute its population, consisting to the Bantu (Konambebe), the Pygmies (Baka), who mainly live by hunting, gathering, subsistence farming, cacao farming, and trade, small animal rearing and sometimes fishing. Gribe is under the influence of an equatorial climate, with an alternation of four distinct annual seasons. The average rainfall is approximately 1500 mm and varies considerably among years [12]. This locality is part of the humid semi-deciduous forest with some elements of the evergreen forest [13]. In terms of hydrography, the Gribe village is watered by the Kwopkwop River, a tributary of the Boumba River, itself tributary of the Ngoko, which is also a tributary of the Great Congo River. The soils encountered in the Gribe village belong to the ferallitic type. Wildlife found presence of 31 large and medium-sized mammal species [14]. In the Gribe village, farming lands result from the felling of forest. The farms are left to fallow depending on the productivity of soil. Agriculture and NTFP collection are the main livelihood activities in the locality. The region has become the target of many forest exploiters in search for valuable species because of its exceptional flora and fauna (Fig. 1).

### Study design

#### Selection of the species

The eight NTFPs species selected for this study were the most economically important species for the area, identified by [5, 6] using the frequency of utilization and commercialization at the village level through a participatory survey with the local population. The following eight species were: *Afrostyrax lepidophyllus, Baillonella toxisperma, Irvingia gabonensis, Panda oleosa, Pentaclethra macrophylla, Ricinodendron heudelotii, Scorodophloeus zenkeri and Tetrapleura tetraptera* (see Additional file 1). The main parts of the species used are roots, barks, pods, fruits, seeds and kernels (Table 1).

**Table 1:** Uses of the selected NTFPs

No.	NTPFs	Trade name	Part uses	Uses
1	*Afrostyrax lepidophyllus*	Garlic tree	Seed, leave and bark	Alimentation, medicinal and fuelwood
2	*Baillonella toxisperma*	Moabi oil	Seed and wood	Alimentation, medicinal, spiritual, construction and fuelwood
3	*Irvingia gabonensis*	Bush mango	Fruits and kernel	Alimentation, medicinal, cosmetics and fuelwood
4	*Panda oleosa*	Bush groundnut	Bark and kernel	Alimentation and medicinal
5	*Pentaclethra macrophylla*	African oil bean	Pod, seed and bark	Alimentation, medicinal, cosmetics, spiritual handicraft and fuelwood
6	*Ricinodendron heudelotii*	Njansang	Kernel and bark	Alimentation, medicinal, cosmetics and fuelwood
7	*Scorodophloeus zenkeri*	Garlic tree	Seed and bark	Alimentation, medicinal and fuelwood
8	*Tetrapleura tetraptera*	Aidan tree	Root, leave and Kernel	Alimentation, medicinal and fuelwood

#### Data collection

**Fig. 2:**
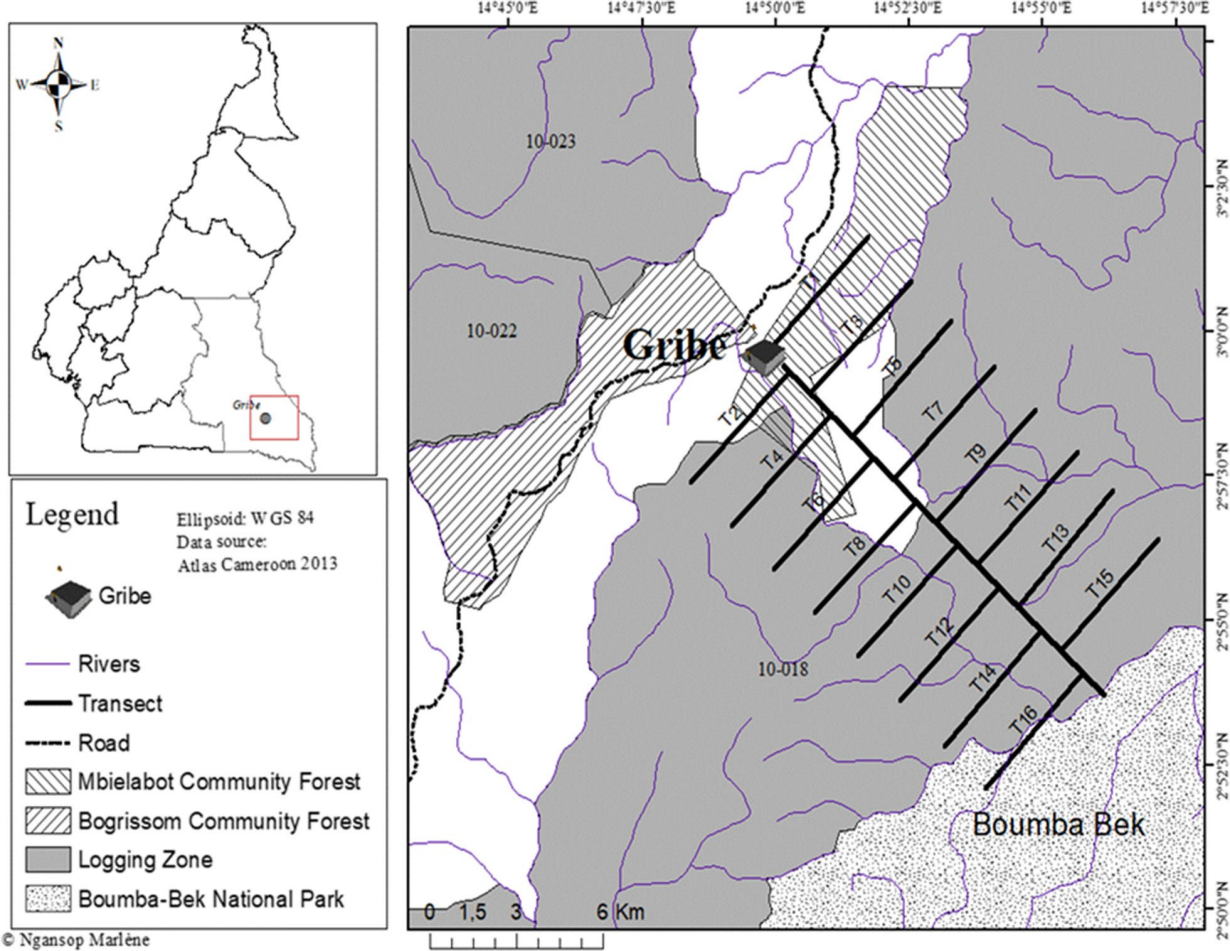
Experimental design

Linear transects were used in this study to sample eight major NTFPs according to [15]. On a 16 km baseline stretching north-west to south-east, 16 transects of 5000 m × 20 m was installed. The equidistance between two consecutive parallel transects was 1 km. The total area sampled was 160 ha (Fig. 2). Along each transect, the determination of habitats types was based on visual assessment of physiognomic and ecological characteristics (see Additional file 2). According to [15] the main parameters taken in account were: dominant tree species, indicator species, open or close canopy, tree density and height, understorey density, topography of the environment, and the origin and degree of disturbance. The determination of habitat types was also based on previous description done by [13]. Along each transect, the GPS coordinates at the start and end points of each habitat type were recorded and used to determine the length and surface area of each habitat type. A total inventory of the individuals of targeted NTFPs was done within 10 m on either sides of each transects.

### Data analysis

The proportion of each habitat type was obtained by calculating the ratio of the transect length occupied by each habitat type to the total lengths of each transect sampled.

The density of species for each habitat type was calculated using the formula: *D *=* n*_*i*_*/S*, where: *D *= stems density (ha^−1^); *n*_*i*_ = number of individuals of species in each habitat type; *S *= total sampling area.

The average density for each NTFP was obtained by summing the density of NTFP in each habitat type and divided by the number of samples. Analysis of variance (ANOVA) and Turkeys test were performed to examine whether NTFP densities varied according to species and in function of habitat types.

## Results

### Diversity of habitats

A total of 13 habitat types were identified based on their physiognomic and physiological characteristics. The most frequent were young secondary forests (32.70%), periodically flooded forests (26.31%), and young Marantaceae secondary forests (19.00%). The least represented were young fallows with *Chromolaena odorata* (0.08%), mid-age fallows (0.15%), food crop fields (0.16%), and old fallows (0.45%) (Table 2).

**Table 2:** Diversity of habitat types in the northern periphery of Boumba-Bek National Park

No.	Habitat type	Distances (m)	Surface area (ha)	Proportion (%)
1	Cacao agroforest	887	1.77	1.11
2	Forest gap	1161	2.32	1.45
3	Food crop field	132	0.26	0.16
4	Swamp	2374	4.75	2.96
5	Periodically flood forest	21,067	42.13	26.31
6	Old secondary forest	7384	14.77	9.22
7	Young secondary forest	26,186	52.37	32.70
8	Young Marantaceae secondary forest	15,212	30.42	19.00
9	Mid-age secondary forest	3641	7.28	4.55
10	Old fallow	357	0.71	0.45
11	Young fallow	1497	2.99	1.87
12	Young fallow with *Chromolaena odorata*	62	0.12	0.08
13	Mid-age fallow	121	0.24	0.15
	Total	80,081	160	100

The proportion of vegetation types was obtained by calculating the ratio of the distance occupied by the habitat type to the total distance of all transects

*CAA* cocoa agroforest, *FOG* forest gap, *FCF* food crop field, *SWP* swamp, *PFF* periodically flooded forest, *OSF* old secondary forest, *YSF* Young secondary forest, *YSF-Ma* young Marantaceae secondary forests, *MISF* mid-age secondary forests, *OLF* old fallow, *YOF* young fallow, *YOF-Co* young fallow with *C. odorata*, *MIF* mid-age fallow

### Density of NTFPs by habitat type

The results showed strong differences in stem densities of NTFP species across habitat types (Table 3).

**Table 3:** Stems density of NTFPs per hectare in different habitat types

Habitat types	Species								Average density
	*A. lepidophyllus*	*B. toxisperma*	*I. gabonensis*	*P. oleosa*	*P. macrophylla*	*R. heudelotii*	*S. zenkeri*	*T. tetraptera*	
CAA	0.11	0	0	0	0.02	0.05	0	0.14	(0.32 ± 1.25)^b^
FOG	1.14	0.01	0.03	0.04	0.38	0.11	0.11	0.03	(1.85 ± 3.07)^b^
FCF	0.1	0	0.01	0	0.06	0	0	0	(0.17 ± 0.65)^b^
SWF	1.57	0	0.03	0.06	0.34	0.18	0.08	0.09	(2.35 ± 6.95)^b^
PFF	5.61	0	0.33	0.5	2.72	0.94	2.31	0.13	(12.53 ± 12.90)^b^
OSF	3.63	0.02	0.11	0.18	0.83	1.71	0.39	0.02	(6.90 ± 13.9)^b^
YSF	13.36	0.03	1.09	0.84	5.28	5.22	0.96	0.31	(27.08 ± 26.89)^a^
YSF-Ma	4.86	0.01	0.23	0.39	1.1	1.68	3.5	0.13	(11.89 ± 21.20)^b^
MISF	1.22	0.01	0.09	0.12	0.36	0.26	0	0.06	(2.11 ± 4.08)^b^
OLF	0	0	0	0.01	0.01	0.01	0.01	0	(0.04 ± 0.11)^b^
YOF	0.41	0	0.03	0.01	0.21	0.15	0	0.03	(0.83 ± 2.83)^b^
YOF-Co	0	0	0.01	0	0	0	0	0	(0.01 ± 0.03)^b^
MIF	0	0	0	0	0	0.03	0	0	(0.03 ± 0.10)^b^
Average density	(32.0 ± 21)^a^	(0.1 ± 0.1)^d^	(1.9 ± 1.6)^cd^	(2.1 ± 1.0)^cd^	(11.3 ± 8.2)^b^	(10.3 ± 18.5)^bc^	(7.4 ± 12.8)^bcd^	(0.9 ± 0.8)^d^	

Average density values with same letter are not significantly different

*CAA* cocoa agroforest, *FOG* forest gap, *FCF* food crop field, *SWP* Swamp, *PFF* periodically flooded forest, *OSF* old secondary forest, *YSF* young secondary forest, *YSF-Ma* young Marantaceae secondary forests, *MISF* mid-age secondary forests, *OLF* old fallow, *YOF* young fallow, *YOF*-*Co* young fallow with *C. odorata*, *MIF* mid-age fallow

*Afrostyrax lepidophyllus* with an average density of 32.0 ± 26.1 stems ha^−1^, was present in 10 habitat types. The highest number of individuals of *A. lepidophyllus* was found in young secondary forests (13.36 stems ha^−1^), periodical flood forests (5.61 stems ha^−1^) and young Marantaceae secondary forests (4.86 stems ha^−1^). Its densities were lower in food crop fields, cocoa agroforests and in fallows.

*Baillonella toxisperma* with an extremely low total density of 0.1 ± 0.1 stems ha^−1^, was present in five habitat types and was most abundant in young and old secondary forests with 0.03 and 0.02 stems ha^−1^, respectively. For mid-age secondary forests, young Marantaceae secondary forests and forest gaps low densities of *B. toxisperma* (0.01 stems ha^−1^) were recorded.

*Irvingia gabonensis* with an average density of 1.9 ± 1.6 stems ha^−1^, was present in 10 habitat types. The highest number of individuals was found in young secondary forests (1.09 stems ha^−1^) followed by periodically flooded forests (0.33 stems ha^−1^) and young Marantaceae secondary forests (0.23 stems ha^−1^). Forest gaps, swamp forests and young fallows each had a density of 0.03 stems ha^−1^. The young fallow with understory dominated by *Chromolaena odorata* and food crop field were habitat types with lowest densities (0.01 stems ha^−1^).

*Panda oleosa* with a total density of 2.1 ± 1.0 stems ha^−1^, was found in nine habitat types. *P oleosa* was absent in cocoa forest, food crop fields, young fallows with understory dominated by *C. odorata*, and in mid-age fallows. Habitat types with high densities of *P. oleosa* individuals were young secondary forests and young Marantaceae secondary forests with densities of 0.84, and 0.39 stems ha^−1^ respectively.

*Pentaclethra macrophylla* had an average density of 11.3 ± 8.2 stems ha^−1^ and was present in 11 habitat types. Young secondary forests, periodically flooded forests, were the habitat types with the highest number of individuals, with densities of 5.28, 2.72 stems ha^−1^ respectively. Old fallows, cocoa agroforests and food crop fields had low densities (0.02–0.06 stems ha^−1^).

*Ricinodendron heudelotii* with an average density of 10.3 ± 18.5 stems ha^−1^, was present in 11 habitat types. The highest number of individuals was found in young secondary forests (5.22 stems ha^−1^) followed by old secondary forests (1.71 stems ha^−1^) and young Marantaceae secondary forests (1.68 stems ha^−1^). The habitat types with low densities of *R. heudelotii* individuals were old fallows (0.01 stems ha^−1^); followed by mid-aged fallows and cocoa agroforest (0.03 and 0.05 stems ha^−1^).

*Scorodophloeus zenkeri* was the fourth most densely represented species at the periphery of Boumba-Bek National Park (7.4 ± 12.8 stems ha^−1^). This species was present in seven habitat types and was found mostly in young Marantaceae secondary forests (3.50 stems ha^−1^) and periodically flooded forests (2.31 stems ha^−1^). Old fallows (0.01 stems ha^−1^) and swamp forests (0.08 stems ha^−1^) were the habitat types with very low densities of individuals.

*Tetrapleura tetraptera* with an average density of 0.9 ± 0.8 stems ha^−1^, was present in nine habitat types. The highest number of individuals was found mainly in young secondary forests (0.31 stems ha^−1^), cocoa agroforests (0.14 stems ha^−1^), young Marantaceae secondary forests and periodically flooded forests (0.13 stems ha^−1^). It was least represented in old secondary forests, young fallows and forest gaps (0.02–0.03) stems/ha) (Table 3).

The ANOVA showed a significant difference (P < 0.05) in the density of NTFP species. It also showed that, for densities of all the eight NTFPs in different habitat types, there was no significant difference between habitat types (P > 0.05) with the exception of young secondary forest where average density was significantly different (P < 0.05) from all other habitat types, and represents the habitat type with the highest number of individuals of all the eight NTFPs (Table 3).

## Discussion

### Diversity of habitats

The northern periphery of Boumba-Bek national park showed several [14] forest habitat types. This diversity is due to high human activities such as forest logging and intensification of agricultural activities. Southeast Cameroon is under logging since 1960 [16]. The Boumba-Bek National Park consist of Forest Management Units (10-018, 10-022, 10-023) and the Mbielabot Community forest where logging activities have led to fragmentation of the forest into several land use types with high proportions of secondary formations. Although the creation of Protected Areas is a cornerstone for nature conservation, they however increase pressure on land for agriculture and contribute to the degradation of the forest [17]. It is also reported that the process of secondarization in tropical forests could also be linked to the internal dynamics of the forest [10].

With regards to habitat type, young secondary forests were the most represented. This could illustrate an advanced level of secondarization in this forest. The process of secondarization of the tropical forest as a result of human disturbance and the action of elephants has already been discussed by several authors [13, 18]. However, high density of individuals of these eight NTFPs was recorded in young secondary forests, evidence that this habitat is favourable to the development of the eight NTFPs selected. The habitats with low occurrence of the targeted species were young fallows dominated by *Chromolaena odorata*. The invasion of *C. odorata* is reported to be the primary factor responsible for the poor regeneration of degraded forests [19, 20]. Environmental factors such as ventilation, humidity and light are parameters that influence the regeneration of species in the forest understory [6]. This can also explain the low availability of individuals in fallows with more closed canopies and choked environments, contrary to open surroundings which are more favourable for growth of plants. It is in this logic that some authors reported that diversity of species is higher in gaps because different microclimates allow for an overall sustained increase in plant establishment and growth due to availability and variation of minerals resource that plant need to growth [21]. Similarly, slash-and-burn agriculture practiced in this zone is one of the parameters that explain the low number of individuals of these NTFPs in cultivated areas. Nevertheless, NTFP availability varies from one habitat type to another and is based on biotic and abiotic factors. Various factors such as biological characteristics, the ability of plant species to tolerate disturbances, as well as anthropic actions, can either limit the availability of NTFPs to restricted ecosystems, or allow them to exist not only in mature and secondary forests, but also in various agroforestry systems such as cacao plantations and mixed croplands [22].

### Species density per habitat type

*Afrostyrax lepidophyllus* was found in almost all habitat types. The wide distribution of this species is thought to be due to seed dissemination in forest by small mammals [6]. *A. lepidophyllus* had a higher density in young secondary forests and in young Marantaceae secondary forests than in mid-age fallows and young fallows dominated by *Chromolaena odorata*. *A. lepidophyllus* appears to be relatively abundant in the different habitat types of the northern periphery of Boumba-Bek National Park. These observations are consistent with previous studies reporting the wide distribution of *A. lepidophyllus* in semi-deciduous forests and in the Dja Wildlife Reserve [23].

*Baillonnella toxisperma* was rare in the several habitat types surveyed. This species has become rare in the forest due to logging activities [24]. Intensive fruit gathering for oil extraction has been reported to affect its regeneration potential [25]. The presence of *B. toxisperma* in fallows, food and cash crops (cocoa, coffee) farms has been justified by the fact that the individuals are often preserved and protected for its multiples uses by the land owners [24]. Even though *B. toxisperma* is a shade tree species, it has been shown that forest gaps are suitable for its growth period [26], yet in this study very few individuals were seen in the open areas. This could be due to an increased use of shifting cultivation in Gribe village.

*Irvingia gabonensis* had a wide distribution and was presented in 10 habitat types. The highest number of individuals for this species was in young secondary forests followed by periodically flooded forests. These results are contrary to results obtained in previous studies conducted in and around the periphery of the Dja Wildlife Reserve, reporting that this species only occur in mature secondary forests [27]. The species was scarcely distributed in the food crop fields and fallows, probably due to the high anthropic activities in these ecosystems.

*Panda oleosa* was present in almost all habitat types, but was distributed scarcely. It was most abundant in young secondary forest and young Marantaceae secondary forest. The distribution of this NTFP in the majority of habitat types in the northern periphery of Boumba-Bek National Park corroborates previous observations that *P. oleosa* is distributed throughout the forest [23].

*Pentaclethra macrophylla* was highly abundant in the northern periphery of Boumba-Bek National Park. This distribution is due to its great fruiting and germination capacities, indicating its wide regeneration potential [6]. High densities for this species were obtained mainly in young secondary forests and in periodically flooded forests. These results are similar to those of previous studies indicating that *P. macrophylla* is a species of tropical secondary forests [28, 29]. The species was sparsely found in cocoa agroforest, food crop fields and fallows. Its low density in these habitats is due to human actions through deforestation and the use of bush fires, which considerably decreases the available potential of the species by destroying seedlings and soil seed banks [9].

*Ricinodendron heudelotii* showed a high density in forest gaps, and mid-age fallows. These habitat types correspond to open environments. This species is classified as a pioneer species in the broader sense, or, more specifically, as a facultative pioneer species [7]. Other authors described it as an opportunistic helophyte species [30]. Thus, its preference for open environments gives it a wide distribution in the aforementioned habitat types. In the Dja Wildlife Reserve, the presence of this species was previously reported for riparian forests, young secondary forests and sparsely-spaced young secondary forests [27]. Dense secondary forests had lower proportions of individuals, providing further evidence that this NTFP species develops preferentially in open habitats. [31], have also mentioned that creation of gaps, can improve the growth of this species.

*Scorodophloeus zenkeri* was seen primarily in young Marantaceae secondary forests, periodically flooded forests, and in young secondary forests. Previous studies had confirmed the presence of this species in young Marantaceae secondary forests and in young secondary forests in Boumba-Bek and Nki National Parks [7] and in the evergreen Atlantic and the Dja forests [23].

*Tetrapleura tetraptera* was mostly seen in young secondary forests, and in cocoa agroforests. [32], have also reported that trees are widespread in tropical forest, especially secondary forest. Although some studies reported a wide distribution of *T. tetraptera* on cultivated lands [23], we encountered it only in cocoa farms and in young fallows. Although [31] revealed that forest gaps are environments favourable for the growth of *T. Tetraptera*, in the present study the species was poorly represented in forest gaps.

### Implications for sustainable management of forest for NTFP production

Forest of the locality of Gribe, have faced great anthropic pressure leading to fragmentation degradation of the forest into several habitats. Today the most represented habitats are young secondary forests, periodically flooded forests and young Marantaceae secondary forests. This degradation results from industrial logging and community exploitation, which leads to impoverishment in NTFP species. It was previously reported that forest loss and fragmentation are among the main drivers of species extinction [33].

In food crop fields and fallows, a small proportion of suited NTFP species was present, which shows that transformation of forest land to others land use types has a negative impact on the availability of the natural resources. An example of this is the species *Baillonella toxisperma* which was completely absent in all cultivated ecosystems. Other species such as *Irvingia gabonensis, Panda oleosa* and *Tetrapleura tetraptera* and were less abundant in the Gribe village forest. This poor abundance results from the slash and burn shifting cultivation frequently used in the village. It is clear that the creation of cultivated lands contributes to deforestation and degradation of forest and negatively affects the abundance of trees species. To overcome this problem, it would be important for populations to: conserve useful plants when clearing forest to create new farm; concentrate on already existing farm land; make use of bio-fertilizers in their farming techniques; intensify and diversify their farms with NTFPs through planting in the agrosystems. This can contribute to reduce the pressure in the forest and favor the attenuation and adaptation to the effects of climate change.

It is very evident that industrial logging causes the deforestation and degradation of forest, reducing the potential of the eight NTFPs considered in this study. To remedy the situation, some authors advocate minimizing of damage to tree species during logging [6]. Although logging causes a lot of damage and reduces the availability of natural resources, it promotes the growth of helophyte species like *Ricinodendron heudelotii*. This domestication operation in open area is also applicable to *B. toxisperma.* [24], already observed the high survival rate of *B. toxisperma* in logging gap environment when compared to canopy cover.

In view of increasing degradation of the forest ecosystems and the more and more increasing request in NTFPs on the Cameroonian and international market, it would be wise to value forest ecosystem management that guarantees the sustainability of the NTFP resources. This management would involve a reduction of slash and burn shifting cultivation, selective cutting of the forest trees during the creation of farms. To ensure the future of NTFP species, it would be essential to promote the domestication of these species particularly in young secondary forest which is the favourable habitat type for the growth of all the eight NTFPs studied, and in agro-systems. This would increase NTFP resource availability and thus contribute to the well-being of local communities in the periphery of Boumba-Bek National Park.

## Conclusion

The northern periphery of the Boumba-Bek National Park is very diverse in terms of habitats, with the predominance of young secondary forests and cultivated ecosystems indicating great fragmentation of the forest. Few NTFPs, species are present in fallows of different ages, in cocoa farms and food crop fields. *Baillonella toxisperma* which is rare in the study site requires domestication intensification to ensure its viability. Young secondary forests represent the main habitat type for all NTFPs selected in this study, and therefore are more favourable for the domestication of all less abundant NTFPs of the study site. While for the helophyte species like *Ricinodendron heudelotii*, their regeneration should be considered in the open environments such as forest gaps. In addition, promoting farmers managed natural regeneration and participatory NTFP domestication in cultivated ecosystems can be considered as viable approaches to conserve diversity among NTFP populations and ensure sustainable supply of these products. To achieve this, research efforts will be needed to improve scientific knowledge on the reproductive biology of these species, their phenology and ecological performance. Although *Afrostyrax lepidophyllus, Pentaclethra macrophylla*, *Ricinodendron heudelotii* and *Scorodophloeus zenkeri* are abundant in this forest, their sustainable management needs to be valorized in order to assure their future availability.

## Additional files

**Additional file 1: Fig. S1.** Fruits, seeds or kernels of some NTFPs: **a** Seeds of *Afrostyrax lepidophyllus*, **b** seeds of *Baillonella toxisperma*, **c** kernels of *Irvingia gabonensis*, **d** seeds of *Panda oleosa*, **e** seeds of *Pentaclethra macrophylla*, **f** seeds of *Ricinodendron heudelotii*, **g** seeds of *Scorodophloeus zenkeri*, **h** seeds of *Tetrapleura tetraptera*.

**Additional file 2: Table S1.** Habitat types characteristics.

## Abbreviations

USD: United States Dollars
NTFP: non timber forest product

## References

[1] Fongnzossie FE, Nkongmeneck B-A, Tsabang N, Nguenang GM (2010). The importance of habitat characteristics for tree diversity in the Mengamé Gorilla Reserve (South Cameroon). Tropics..

[2] Sonké B, Couvreur T (2014). Tree diversity of the Dja Faunal Reserve, Southeastern Cameroon. Biodivers Data J.

[3] Awono A, Levang P (2018). Contribution of environmental products to the household economy in Cameroon: essential, complementary or trivial?. Forest Res Eng Int..

[4] Jimoh SO, Amusa TO, Azeez IO (2013). Population distribution and threats to sustainable management of selected non-timber forest products in tropical lowland rainforests of south western Nigeria. J For Res.

[5] Hirai M (2014). Agricultural land use, collection and sales of non-timber forest products in the Agroforest Zone in Southeastern Cameroon. Afr Stud Monogr Suppl Issue..

[6] Fongnzossie FE, Ngansop TM, Zapfack L, Kemeuze VA, Sonwa DJ, Nguenang GM, Nkongmeneck B-A (2014). Density and natural regeneration potential of selected commercial non-timber forest products in the semi-deciduous rainforest of southeastern Cameroon. Afr Stud Monogr.

[7] Nkongmeneck BA. The Boumba-Bek and Nki forest reserves: botany and ethnobotany.WWF Cameroon. Research report. 1999. p. 146.

[8] Souare K. Gestion intégrée des espèces ressources clés des Produits Forestiers Non Ligneux végétaux du parc national du Mbam et Djerem et sa périphérie (Cameroun). Thèse de Doctorat Ph. D Université de Yaoundé I. 2013. p. 97.

[9] Mouamfon M, Guedje NM, Pepainyiene I, Zapfack L, Ngueguim RJ, Lejoly J (2015). *Pentaclethra macrophylla* Benth dans la forêt communautaire de Payo (Est Cameroun): inventaire, productivité et commercialisation. Int J Biol Chem Sci.

[10] Nguenang GM, Nkongmeneck B-A, Gillet J-F, Vermeulen C, Dupain J, Doucet J-L (2010). Etat actuel de la sécondarisation de la forêt en périphérie nord de la Réserve de biosphère du Dja (Sud-est Cameroun): influences des facteurs anthropiques passés et des éléphants. Int J Biol Chem Sci..

[11] Toda M (2014). People and social organizations in Gribe, Southeastern Cameron. Afr Study Monogr..

[12] Yasuoka H (2006). The sustainability of duiker (*Cephalophus* spp.) hunting for the Baka hunter-gatherers in southeastern Cameroon. Afr Study Monogr..

[13] Letouzey R. Notice de la carte phytogéographique du Cameroun au 1:500000. Domaine de la forêt dense humide semi-caducifoliée. Institut internationale de la végétation, Toulouse, France. 1985. p. 240.

[14] Bobo KS, Kamgaing TOW, Ntumwel BC, Kagalang D, Kengne PNJ, Ndengue SML, Badjeck MMN, Aghomo FFM (2014). Species richness, spatial distributions and densities of large- and medium-sized mammals in the northern periphery of Boumba-Bek National Park, Southeastern Cameroon. Afr Study Monogr..

[15] White L, Edwards A (2000). Conservation en forêt pluvial Africaine: méthodes de recherche.

[16] Mouncharou G, et Ngnegue UPR. Contribution à la mise en place du système de suivi de la lutte anti-braconnage et de l’abattage de l’éléphant dans la région du Sud-Est Cameroun. WWF jengi-SE project. 2001. p. 47.

[17] Beatrice AF, Tabot TP, Bakia M-A, Awah CC (2018). Patterns of land-use change and current vegetation status in peri-urbanforest reserves: the case of the Barombi Mbo Forest Reserve. Cameroon, Geol Ecol Landsc..

[18] Nkongmeneck B-A, Amsallem NR, Drouineau SI (1998). Processus de secondarisation en forêt dense humide camerounaise. La Gestion des Forêts Denses Africaines Aujourd’hui.

[19] Swaine MD, Agyeman VK, Kyere B, Orgle TK, Thomson J, Veenendaal EM. Ecology of forest trees in Ghana. ODA Forestry Series No. 7, London, UK. 1997. p. 76.

[20] Honu YAK, Dang QL (2000). Distribution and species composition of seeds and seedlings of trees and competing vegetation in Ghana. For Ecol Manag.

[21] McCarth YJ (2001). Gap dynamics of forest trees: a review with particular attention to boreal forests. Environ Rev..

[22] Pulido MT, Caballero J (2006). The impact of shifting agriculture on the availability of non-timber forest products: the example of Sabal yapain the Maya lowlands of Mexico. For Ecol Manag..

[23] Vivien J, Faure JJ (2011). Arbres des forêts denses d’Afrique centrale.

[24] Mbolo M. Etude détaillée sur la collecte et l’analyse des données statistiques des Produits forestier Non-Ligneux au Cameroun. 2002. p. 70.

[25] Schneemann J (1995). Exploitation of Moabi in the humid dense forests of Cameroon: harmonization and improvement of two conflicting ways of exploitation of the same forest resource. BOS Newslett..

[26] Kouadio YL, Doucet J-L (2009). Étude du comportement de *Baillonella toxisperma* Pierre (moabi) dans les trouées d’abattage enrichies. Biotechnol Agron Soc Environ.

[27] Nguenang GM. Secondarisassions et dynamique cicatricielle de la forêt du Dja (Est-Cameroun): application à l’aménagement des formations secondaires. Thèse de Doctorat Ph.D Université de Yaoundé I. 2013. p. 239.

[28] Oboh G. *Pentaclethra macrophylla* Benth. In: Egetable Oils/Oléagineux, Van der Vossen, Kamilo MGS (éds). PROTA 14: Wageningen, Hollande; [En ligne]. 2007. http://www.database.prota.org/PROTAhtml/. Consultéle 25 mars 2018.

[29] Plenderleith K (2000). *Ricinodendron heudelotii:* a state of knowledge report undertaken for the Central African Regional Program for the environment.

[30] Gwamashi ET. Inventaire des espèces ligneuses locales pour le reboisement à des fins énergétiques. Mémoire Master, Université de Kinshasa. 2009. http://www.memoireonline.com. Consulté le 20 janvier 2018.

[31] Egbe EA, Tsamoh TT (2018). Vegetation studies of Non-Timber Forest Products (NTFPs) at three sites with varying levels of anthropogenic disturbances in the Southern Bakundu Forest Reserve, Cameroon. J Ecol Nat Environ..

[32] Orwa CA, Mutua KR, Jamnadass RS, Anthony. Agroforestry Data base: a tree reference and selection guide version 4.0. 2009. http://www.worldagroforestry.org/sites/treedbs/treedatabases.asp. Consulté le 10 Sept 2018.

[33] Hermes C, Döpper A, Schaefer MH, Segelbacher G (2016). Effects of forest fragmentation on the morphological and genetic structure of a dispersal-limited, endangered bird species. Nat Conserv..

