# Supplementary figures and images for: Using transect sampling to determine the distribution of some key non-timber forest products across habitat types near Boumba-Bek National Park, South-east Cameroon
Source: BMC Ecol. 2019 Jan 22;19:3. doi: 10.1186/s12898-019-0219-y (PMC6343285; doi:10.1186/s12898-019-0219-y)

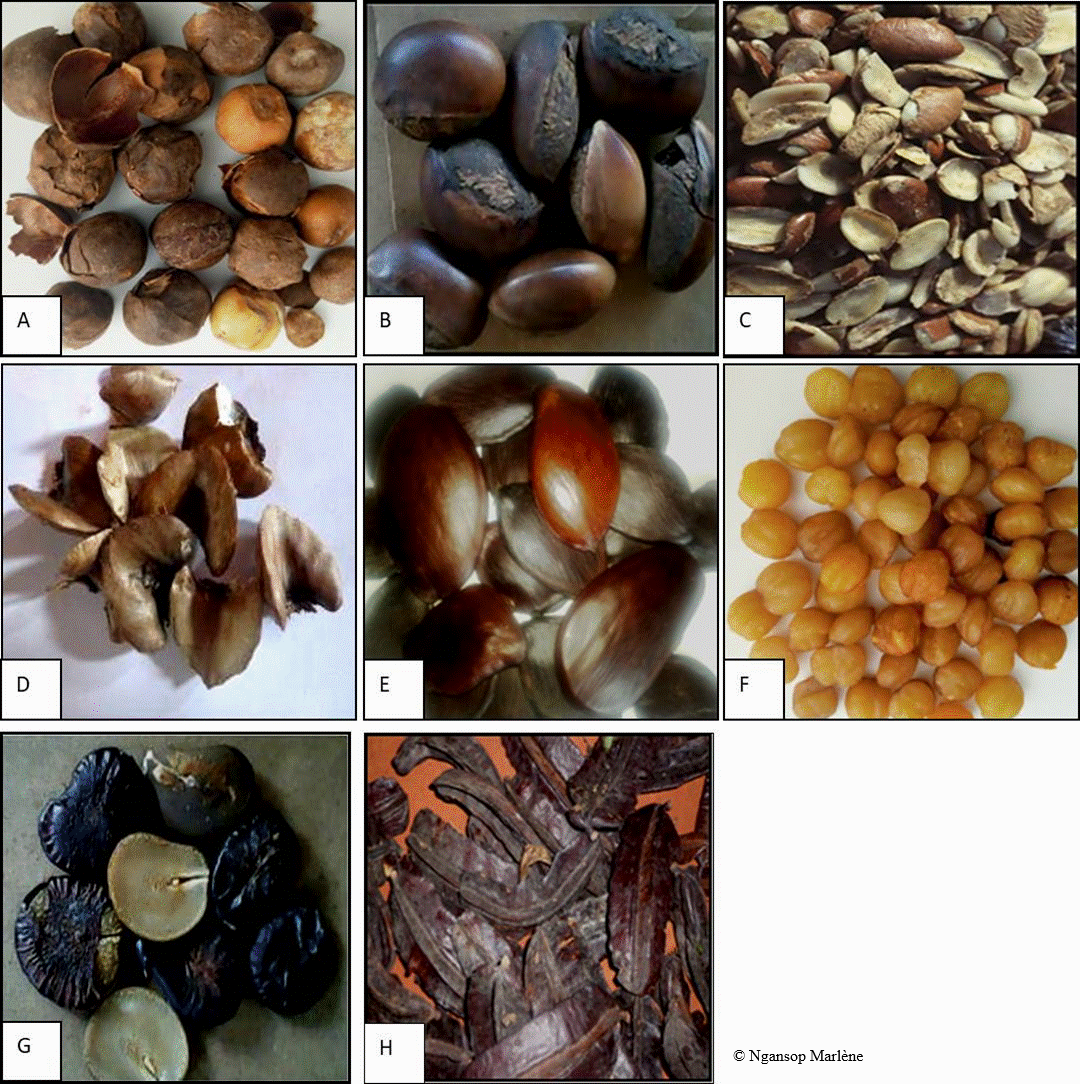

Supplement: Supplementary file 1 — Additional file 1: Fig. S1. Fruits, seeds or kernels of some NTFPs: a Seeds of Afrostyrax lepidophyllus, b seeds of Baillonella toxisperma, c kernels of Irvingia gabonensis, d seeds of Panda oleosa, e seeds of Pentaclethra macrophylla, f seeds of Ricinodendron heudelotii, g seeds of Scorodophloeus zenkeri, h seeds of Tetrapleura tetraptera. [file 12898_2019_219_MOESM1_ESM.tiff]
