# Supplementary material for: Using transect sampling to determine the distribution of some key non-timber forest products across habitat types near Boumba-Bek National Park, South-east Cameroon
Source: BMC Ecol. 2019 Jan 22;19:3. doi: 10.1186/s12898-019-0219-y (PMC6343285; doi:10.1186/s12898-019-0219-y)
Supplement: Supplementary file 2 — Additional file 2: Table S1. Habitat types characteristics. [file 12898_2019_219_MOESM2_ESM.docx]

Table S1. Habitat types characteristics

| N° | Habitat types | Characteristics |
| --- | --- | --- |
| 1 | Cacao agroforest | Dominated with the presence *Theobroma cacao.* |
| 2 | Forest gap | Open areas in the forest which could originate from fall of trees. |
| 3 | Food crop field | Farmland in which food crops (cassava, plantain, cocoyam, maize, bean) are cultivated. |
| 4 | Swamp | Low land areas, constantly flooded, or flooded all year round. |
| 5 | Periodically flood forest | Low land forest areas, often flooded in the wet seasons, thick dark-brown coloured soil. |
| 6 | Old secondary forest | Characterized with a primary forest appearance, presence of the big diameter trees and undergrowth almost completely open of easy access and an also open canopy (semi-deciduous forest). |
| 7 | Young secondary forest | Presence of legacies that suggested previous anthropic interventions such as timber logging. The massive abundance of light demanding tree species e.g. *Musanga cercropoïdes; Trema orientalis etc.* |
| 8 | Young Marantaceae secondary forest | Dominated with the presence of Marantaceae species as: *Haumania dancklemaniana, Marantocloa spp., Megaphrynium macrostachyum; Sacrophrynium brachystachys etc.* |
| 9 | Mid-age secondary forest | Less dense undergrowth and vegetation characterized by the disappearance of some helophyte species. Fairly elevated canopy |
| 10 | Old fallow | Chocked environment, abundance of herbaceous plants, upper to 10years of fallow |
| 11 | Young fallow | Chocked environment, abundance of herbaceous plants of less than 5 years of fallow |
| 12 | Young fallow with *Chromolaena odorata* | Chocked environment, abundance of herbaceous plants, and dominance of *C. odorata* of less than 5 years of fallow |
| 13 | Mid-age fallow | Chocked environment, abundance of herbaceous plants, between to 5-10 years of fallow |
